# Andrographis Paniculata (Burm. F.) Flavonoid Compound and Prevention of Diabetic Retinopathy

**DOI:** 10.18502/jovr.v19i1.15435

**Published:** 2024-03-14

**Authors:** Ramzi Amin, Muhammad Apriliandy Shariff, Petty Purwanita, Mgs Irsan Saleh

**Affiliations:** ^1^Department of Ophthalmology, Faculty of Medicine, Universitas Sriwijaya/Dr Moh Hoesin General Hospital, Palembang, Indonesia; ^2^Department of Pharmacology, Faculty of Medicine, Universitas Sriwijaya, Palembang, Indonesia

**Keywords:** Andrographis paniculata, Antioxidants, Diabetic Retinopathy, Plant Extracts, Retinal Vessels

## Abstract

**Purpose:**

To explore the effect of the flavonoid compounds of *Andrographis paniculata *by evaluating the glycemic profile, oxidative process, and inflammatory values in rats with diabetic retinopathy (DR).

**Methods:**

An extract of *A. paniculata* was macerated with ethanol which yielded flavonoid compounds. Streptozotocin was utilized to induce diabetes mellitus in male Wistar rats. Vucetic's methods were used to evaluate the retinal vessel diameters. Antioxidant parameters and inflammatory cytokines were assessed in retinal tissue.

**Results:**

A funduscopic examination revealed some alterations in the retinal veins. In comparison to the DR group with no treatment, the diameter of the retinal vessels in the DR group that was treated with the flavonoid component of the *A. paniculata* extract (FAP) at doses of 20 and 40 mg/kg body weight (BW) was significantly smaller (*P* < 0.05). The DR treatment groups administered with FAP at doses of 20 and 40 mg/kg BW had a greater ability to reduce TNF-alpha and VEGF levels as compared to the DR rats without treatment (*P* < 0.05), Glutathione, superoxide dismutase (SOD), and catalase levels were increased after receiving FAP at doses of 20 and 40 mg/kg BW (*P* < 0.05).

**Conclusion:**

Administration of doses of 20 and 40 mg/kg BW of the *A. paniculata's *flavonoid compoundsimproved DR in rats via retinal vessel diameter reduction, TNF- α and VEGF level reduction, and increasing antioxidants, SOD, catalase, and glutathione.

##  INTRODUCTION

Diabetic retinopathy (DR) is the most common retinal microvascular disease and a leading cause of visual impairment and blindness on a global scale.^[1,2]^ The elevation of reactive oxidative species (ROS) plays a significant role in the pathogenesis of DR.^[3]^ Diabetes mellitus leads to heightened levels of oxidative stress, which is observed in a wide range of end organs, including the retinal arterioles. Oxidative stress leads to the generation of a series of ROS and enhances the activation of a cascade of proinflammatory mediators, including tumor necrosis factor (TNF-α) and vascular endothelial growth factor (VEGF).^[4]^ These mediators activate the process of increasing the expression of adhesion molecules on endothelial cells and leukocytes. The occurrence of leukostasis has been ascribed to the obstruction of blood vessels and the disruption of microcirculation, particularly in the end organs.^[5]^ The initial histological alterations observed in DR involve specific damage to pericytes, a rise in the thickness of the capillary basement membrane, heightened capillary permeability, and formation of microaneurysms. Structural and functional alterations are accompanied by microvessel blockage, neovascularization, and retinal cell degeneration.^[4,5]^

The retina is highly prone to the oxidative process, which is mainly due to its high content of polyunsaturated fatty acids, high oxygen requirement, glucose oxidation, and long exposure to ultraviolet light.^[6]^ In particular, elevated glucose levels initiate a series of physiological mechanisms, including the aggregation of advanced glycation end-products (AGEs), activation of protein kinase-C (PKC), and heightened metabolic activity in the polyol and hexosamine pathways. These activities all contribute to the induction of oxidative stress. The elevation of ROS is expected to induce DNA fragmentation, leading to the formation of polyadenosine-diphosphate (ADP), the activation of ribose polymerase, and the inhibition of glyceraldehyde 3-phosphate dehydrogenase. This leads to glycolytic metabolites accumulation that can trigger AGE formation and activation of PKC, polyols and hexosamine pathway, which are known to contribute to the pathogenesis of DR. Oxidative stress creates a propagation cycle, leading to increased ROS and activation of pathways closely associated with DR development.^[6]^

Currently, there is no effective therapeutic modality for the prevention of DR. The vast biological resources present in Indonesia provide promise for the exploration and development of novel therapeutic approaches. It is widely recognized that plant species containing antidiabetic compounds have the potential to serve as a valuable source for the development of innovative oral hypoglycemic medications.^[7,8]^

*Andrographis paniculate (AP)*, which is a herbal plant widely grown in Southeast Asian countries including Indonesia, is often used in traditional recipes or as a treatment for various diseases. Several studies have shown the AP extract's ability to reduce blood glucose levels in diabetic rats.^[9,10,11]^ The high flavonoid content in AP can potentially overcome oxidative stress activity in cells.^[12]^ The build-up of advanced glycation end products (AGEs) in retinal cells and tissues promotes the start of oxidative stress in these cellular and tissue environments. This study represents the investigation into the impact of the AP extract on inhibiting the progression of DR in a rat model, via hypoglycemic, antioxidant, and anti-inflammatory pathways.

##  METHODS

### Animals and Diabetes Induction

This study employed an experimental research methodology, specifically utilizing a posttest control group design. Thirty male Wistar rats (Rattus norvegicus) weighing between 220 and 250 gr were obtained from the Eureka Laboratory in Palembang, Indonesia. All rats were housed in individual cages with standardized conditions, including a 12-hour light/dark cycle, ambient temperatures of 20–22ºC, humidity around 50%, and free access to available liquid and food. The diabetic rats were sorted into four groups receiving treatment and one untreated group. Group 1 received aquadest 1 mL, groups 2, 3, and 4 each received FAP suspension of 10, 20, and 40 mg/kg body weight (BW) intragastric, respectively, and group 5 was the normal control (untreated group). The rats in this study were administered streptozotocin (STZ) at a dosage of 45 mg/kg BW to establish a model of diabetes mellitus.^[13,14,15]^ Blood glucose levels were evaluated at the onset of diabetes mellitus induction and 24 hours following the administration of STZ. Diabetes mellitus in rats was defined as blood sugar levels exceeding 200 mg/dL. The blood glucose examination was performed by the glucose oxidase–peroxidase procedure. Blood samples were taken through the distal tail using a blood lancet (Accu-chek Softclix, Roche Diagnostics, India). Hemoglobin A1c (HbA1c) was assessed using glycosylated hemoglobin kit (Biosystems S.A., Costa Brava 30).

The euthanasia process was executed by administering a pentobarbital overdose. The rat's eyeballs were evacuated, and retinal tissue was separated and preserved in liquid nitrogen for evaluation. All experimental procedures were conducted in conformity with the Association for Research in Vision and Ophthalmology (ARVO) guidelines for the care and use of laboratory animals, and were approved by the medical research ethics committee of the Medical Faculty, Universitas Sriwijaya (Ref. No. 089-2021).

### Plant Authentication

This investigation utilized AP leaves harvested in June 2021 in South Sumatera, Indonesia. The Botany Department, Science Faculty, Universitas Sriwijaya, Indonesia, identified the leaves. The identification of the plant specimen (No. 2021/75) is preserved at the Biology Department, Medical Faculty, Universitas Sriwijaya.

### Preparation of Flavonoid Compounds of A. paniculata

*Andrographis paniculata* (AP) leaves were washed and cleaned with running water. Next, the AP leaves were dried by heating in an oven (temperature 60ºC). Then, the AP leaves were refined to obtain AP simplicia. The extraction of AP simplicia was performed using the maceration technique, employing 96% ethanol as the solvent. The extraction process was carried out for a total duration of 3 consecutive cycles, each lasting 24 hours. The ratio of AP simplicia to solvent used was 1:10. The separation between the macerate and the dregs was then completed. The AP macerate was then used to complete the process of isolation of the flavonoid compound. The extracted filtrate was hydrolyzed using 2N HCl in a ratio of 1:1 and then refluxed for 2 hr. The filtrate was then fractionated using ethyl acetate. The ethyl acetate fraction was taken and evaporated to a certain volume. The fraction was then spotted with a band model on the stationary phase of Whatman-1 paper and eluted with 15% acetic acid as the mobile phase. Yellow spots with similar retardation or retention factor (Rf) value and evaporated by ammonia were accumulated and extracted with methanol. The filtrate was evaluated for the flavonoid aglycones component by spectrophotometry at a wavelength of 425 nm compared to quercetin. Subsequently, the mobile phase was substituted with a mixture of Butanol-Acetic Acid-water (BAW) in a proportion of 4-1-5, while the stationary phase was replaced with silica gel F254.

### Preparation of Flavonoid Compounds of A. paniculata (FAP) Oral Suspension

The flavonoid compounds derived from *A. paniculata* (FAP) exhibits solubility in water, making it suitable for the preparation of an aqueous suspension intended for oral administration. The extracts were grinded into a fine powder using a solution containing 0.3% tween 80. Subsequently, a solution of hydroxypropyl methylcellulose (HPMC) at a concentration of 0.25% was continuously mixed to achieve the desired final volume. Hydroxypropyl methylcellulose serves as a viscoelastic agent within suspensions.

### Fundus Photo Assessment

Tropicamide 1% (MydriacylⓇ    ) was administered for pupil dilatation. The fundus photos were shot weekly with a Nikon camera attached on a slit lamp with 16 × magnification plus a 90D lens located anterior to the rat's eye. HPMC 0.7% eye drops were administered repeatedly for cornea hydration. Retinal vessel diameter was estimated using the Vucetic's method.^[16]^ Retinal vessel diameter was assessed at the three most conspicuous locations at the same distance from the center. Three independent investigators carried out the evaluation. The mean of the three blood vessel diameter measurements was set as the retinal vessel diameter.

### Antioxidant Activity Evaluation

The activity of glutathione (GSH) was examined by Moron et al technique.^[17]^ The assessment of superoxide dismutase (SOD) activity was conducted following the procedure outlined by Misra et al.^[18]^ Aebi's method was used to evaluate catalase (CAT) activity.^[19]^ Sample protein assessment was carried out using Lowry procedure.^[20]^ All evaluations were examined twice.

### TNF-alpha and VEGF Evaluations

TNF-alpha levels in the retina were examined using the enzyme-linked immunosorbent assay (ELISA) technique. The ELISA kit from Cloud Clone (Hangzhou, China) was employed for this purpose, following the instructions provided in the accompanying handbook. Retinal VEGF levels were assessed using the ELISA kit obtained from Ray Biotech Inc., located in Georgia, USA. The experimental procedure involved conducting measurements within a 100 mL retinal homogenate. The measurements were conducted twice.

### Statistical Analysis

The data analysis was conducted utilizing the SPSS version 25.0 program. Data were presented in the form of mean values and standard deviations. A one-way analysis of variance (ANOVA) was used to compare each group, followed by a Bonferroni post hoc test.

### Ethics Approval

The treatment of experimental animals in this study has been approved by the medical research ethics committee of the Faculty of Medicine, Universitas Sriwijaya (No. 089-2021).

**Figure 1:**
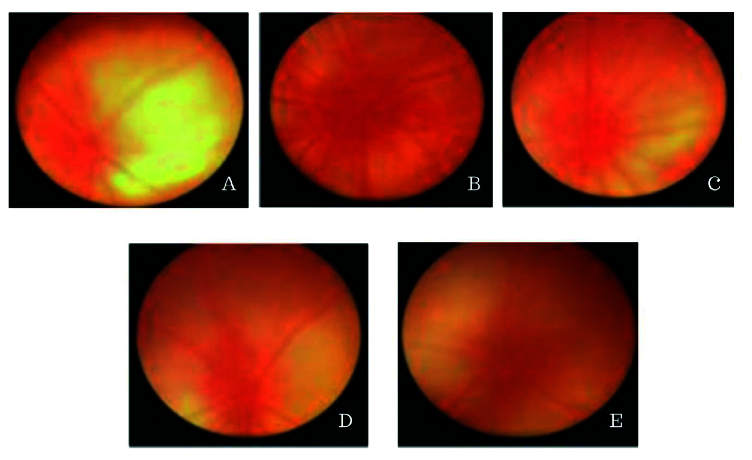
Fundus photographs of rats were taken following various treatments: (A) Normal group; (B) Group with diabetic retinopathy (DR); (C) Group with DR and administered with FAP at dosage 10 mg/kg BW; (D) Group with DR and administered with FAP at dosage 20 mg/kg BW; (E) Group with DR and administered with FAP at dosage 40 mg/kg BW. BW, body weight; DR, diabetic retinopathy; FAP, flavonoid compound of *A. paniculata*.

**Figure 2:**
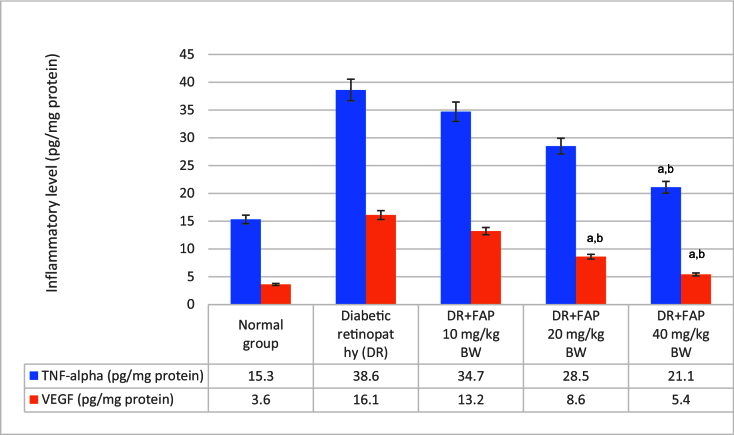
The concentrations of TNF-α (shown by the color blue) and VEGF (represented by the color orange) in the retinal tissue of rats were assessed following a particular course of treatment. ${\;}^{a}$ *P* < 0.05 in contrast to diabetic retinopathy group; ${\;}^{b}$ *P* < 0.05 in contrast to normal group. DR, diabetic retinopathy; FAP, flavonoid compound of *A. paniculata*; TNF, tumor necrosis factor; VEGF, vascular endothelial growth factor.

**Table 1:** The impact of the flavonoid compound derived from *A. paniculata* on glycemic indices following administration.


	**Body weight (gr) ± SD**	**Blood glucose (mg/dL) ± SD**	**% HbA1c ± SD**
Normal group	299.6 ± 26.5	98.8 ± 2.3	4.6 ± 0.5
Diabetic Retinopathy (DR)	279.9 ± 23.5	599.7 ± 37.3	8.4 ± 0.6
DR + FAP 10 mg/kg BW	279.2 ± 25.8 ${\;}^{a}$	577.9 ± 34.9 ${\;}^{b}$	7.8 ± 0.7 ${\;}^{b}$
DR + FAP 20 mg/kg BW	284.8 ± 27.6 ${\;}^{ab}$	379.8 ± 21.5 ${\;}^{ab}$	6.9 ± 0.5 ${\;}^{ab}$
DR + FAP 40 mg/kg BW	291.8 ± 27.3 ${\;}^{ab}$	308.6 ± 21.8 ${\;}^{ab}$	5.7 ± 0.7 ${\;}^{ab}$
	
	
${\;}^{a}$ *P* < 0.05 in contrast to diabetic retinopathy group; ${\;}^{b}$ *P* < 0.05 in contrast to normal group. BW, body weight;DR, diabetic retinopathy; FAP, *A. paniculata *flavonoid compound; SD, HbA1c, hemoglobin A1C standard deviation			

**Table 2:** The impact of FAP on antioxidant markers following intervention.


	**Superoxide dismutase (IU/mg protein) ± SD**	**Catalase (IU/mg protein) ± SD**	**Glutathione (nmol/mg protein) ± SD**
Normal group	19.6 ± 2.5	4.8 ± 1.3	25.6 ± 1.5
Diabetic retinopathy (DR)	9.9 ± 1.5	1.7 ± 0.3	14.4 ± 2.6
DR + FAP 10 mg/kg BW	10.2 ± 1.8 ${\;}^{b}$	1.9 ± 0.9 ${\;}^{b}$	15.1 ± 1.7 ${\;}^{b}$
DR + FAP 20 mg/kg BW	14.8 ± 1.6 ${\;}^{ab}$	2.8 ± 0.5 ${\;}^{ab}$	18.9 ± 0.5 ${\;}^{ab}$
DR + FAP 40 mg/kg BW	17.8 ± 1.3 ${\;}^{ab}$	3.6 ± 0.8 ${\;}^{ab}$	20.7 ± 0.7 ${\;}^{ab}$
	
	
${\;}^{a}$ *P* < 0.05 in contrast to diabetic retinopathy group; ${\;}^{b}$ *P* < 0.05 in contrast to normal group. DR, diabetic retinopathy; FAP, *A. paniculata *flavonoid compound; SD, standard deviation BW, body weight			

##  RESULTS

In this study, we evaluated blood glucose levels and HbA1c to assess DR induction in rats. At the completion of the treatment, it was observed that the diabetic rats exhibited significantly higher blood glucose levels as compared to the normal nondiabetic rats (*P* < 0.05). The equal results were shown in the HbA1c value examination. The HbA1c levels in the group of rats with diabetes were substantially greater as compared to the normal group (*P* < 0.05). In the FAP 20 and 40 mg/kg BW groups, the HbA1c value exhibited a statistically significant rise (*P* < 0.05) when compared to the normal rats. However, the HbA1c value in the FAP groups remained significantly lower (*P* < 0.05) than that of the diabetic rats with no treatment [Table 1].

### Fundus Photo Assessment

The funduscopic photographs exhibited various modifications in the diameter of the retinal vessels, as depicted in Figure 1. The mean retinal vascular diameter in the normal and DR groups was determined to be 51.9 ± 4.7 and 60.2 ± 4.5 pixels, respectively. Likewise, it was observed that the average vessel diameter in the DR + FAP 20 and 40 mg/kg BW group demonstrated a considerably greater decrease compared to the DR group with no treatment (*P* < 0.05). Concurrently, the mean retinal vessel measurements in the DR + FAP group of 10, 20, and 40 mg/kg BW were 59.5 ± 3.4; 56.8 ± 3.9; and 53.2 ± 3.8 pixels, respectively.

### TNF-alpha and VEGF Evaluations

FAP could potentially reduce TNF-alpha and VEGF values, as shown in Figure 2. The DR treatment groups administered with FAP at doses of 20 and 40 mg/kg BW had a greater ability to reduce TNF-alpha levels as compared to the DR rats without treatment (*P* < 0.05).In addition, the administration of FAP at doses of 20 and 40 mg/kg BW demonstrated significant potential in reducing VEGF levels when compared to the rats with DR where no treatment was administered (*P* < 0.05).

### Antioxidant Activity Evaluation

Table 2 shows the potential of FAP in increasing antioxidants, SOD, CAT, and GSH. The groups administered with DR + FAP at doses of 20 and 40 mg/kg BW exhibit a significant increase in SOD and CAT levels when compared to the DR group (*P* < 0.05). The results indicate that the administration of FAP at doses of 20 and 40 mg/kg BW leads to a more pronounced increase in GSH levels compared to the DR rats with no treatment (*P* < 0.05).

##  DISCUSSION

Prior research established a positive correlation between the broader width of retinal arterioles and the occurrence and advancement of DR.^[21,22,23,24]^ Previous studies also revealed that retinal vessel caliber assessment can predict the prognosis related to microvascular complications, including retinopathy.^[23,24]^ In this study, we observed significantly greater retinal vessel dilation in diabetic rats compared to normal rats. FAP treatment resulted in a reduction in retinal vessel caliber compared with the untreated diabetic rats.

Based on the cytokine proinflammatory evaluation, the TNF-alpha gene affected the DR mechanism. Polymorphism of the TNF-alpha gene has been related to the risk of DR.^[25]^ The production of adhesion molecules in endothelial cells is stimulated by TNF-alpha through the activation of the nuclear factor-κB (NF-κB). Therefore, the activation of NF-κB leads to an increase in the expression of the cyclooxygenase-2 (COX-2) enzyme.^[26]^ The induction of COX-2 can also be provoked by glycosylation products. The current study revealed increased levels of TNF-alpha in the retinas of diabetic rats as compared to the normal group [Figure 2].

In contrast, an additional investigation provided support for the notion that levels of retinal TNF-alpha were notably higher in the group of individuals with diabetes.^[27]^ The present study revealed that the treatment of the flavonoid compound of *A. paniculata* (FAP) could lower TNF-alpha levels in DR rats [Figure 2]. This study also presents VEGF levels elevation in the DR group [Figure 2].

VEGF is a proinflammatory cytokine that plays an essential role in the processes of neovascularization and vascular permeability, ultimately leading to retinal vessel damage.^[28]^ Several studies revealed elevated VEGF in DR conditions.^[27,28]^ Moreover, the inhibition of VEGF activity serves to limit the progression of vascular damage in the case of DR. The findings of our current investigation have substantiated the capacity of FAP to reduce levels of VEGF in an animal model of DR [Figure 2].

In DR, antioxidant enzymes (SOD, CAT, and GSH), which act as ROS scavengers and perform homeostasis maintenance of the reduction–oxidation process, are lowered in the retina.^[29]^ Furthermore, intracellular GSH plays a crucial role in enhancing cellular strength, as it is arguably the major defense system within cells. GSH has the capability to function as a scavenger of ROS and maintain equilibrium in the intracellular reduction–oxidation state. In the context of DR, it has been observed that the levels of antioxidant enzymes are diminished, while the functionality of metabolic enzymes is impaired. The results of our investigation indicate that animal models with DR exhibited a reduced level of GSH as well as decreased activity of antioxidant enzymes, such as SOD and CAT, as shown in Table 2. The observed variations in antioxidant levels exhibit similarities to findings reported in previous investigations. The administration of FAP resulted in a slight increase in the levels of SOD, CAT, and GSH, similar to those observed in the normal group.

In summary, the flavonoid compounds of *A. paniculata *administered in dosages of 20 and 40 mg/kg bodyweight improved the condition of DR in the animal models via retinal vessel diameter reduction, TNF- α and VEGF level reduction, and increasing antioxidants, SOD, CAT and GSH levels.

##  Financial Support and Sponsorship

None.

##  Conflict of Interest

None.
